# NanoDeep: a deep learning framework for nanopore adaptive sampling on microbial sequencing

**DOI:** 10.1093/bib/bbad499

**Published:** 2024-01-06

**Authors:** Yusen Lin, Yongjun Zhang, Hang Sun, Hang Jiang, Xing Zhao, Xiaojuan Teng, Jingxia Lin, Bowen Shu, Hao Sun, Yuhui Liao, Jiajian Zhou

**Affiliations:** Dermatology Hospital, Southern Medical University, Guangzhou, China; Dermatology Hospital, Southern Medical University, Guangzhou, China; Dermatology Hospital, Southern Medical University, Guangzhou, China; Dermatology Hospital, Southern Medical University, Guangzhou, China; Li Ka Shing Institute of Health Sciences, The Chinese University of Hong Kong, Prince of Wales Hospital, Shatin, New Territories, Hong Kong SAR, China; Department of Chemical Pathology, The Chinese University of Hong Kong, Prince of Wales Hospital, Shatin, New Territories, Hong Kong SAR, China; Dermatology Hospital, Southern Medical University, Guangzhou, China; Dermatology Hospital, Southern Medical University, Guangzhou, China; Dermatology Hospital, Southern Medical University, Guangzhou, China; Li Ka Shing Institute of Health Sciences, The Chinese University of Hong Kong, Prince of Wales Hospital, Shatin, New Territories, Hong Kong SAR, China; Department of Chemical Pathology, The Chinese University of Hong Kong, Prince of Wales Hospital, Shatin, New Territories, Hong Kong SAR, China; Dermatology Hospital, Southern Medical University, Guangzhou, China; Dermatology Hospital, Southern Medical University, Guangzhou, China

**Keywords:** adaptive sampling, machine learning, nanopore sequencing, convolutional neural network, metagenomic sequencing

## Abstract

Nanopore sequencers can enrich or deplete the targeted DNA molecules in a library by reversing the voltage across individual nanopores. However, it requires substantial computational resources to achieve rapid operations in parallel at read-time sequencing. We present a deep learning framework, NanoDeep, to overcome these limitations by incorporating convolutional neural network and squeeze and excitation. We first showed that the raw squiggle derived from native DNA sequences determines the origin of microbial and human genomes. Then, we demonstrated that NanoDeep successfully classified bacterial reads from the pooled library with human sequence and showed enrichment for bacterial sequence compared with routine nanopore sequencing setting. Further, we showed that NanoDeep improves the sequencing efficiency and preserves the fidelity of bacterial genomes in the mock sample. In addition, NanoDeep performs well in the enrichment of metagenome sequences of gut samples, showing its potential applications in the enrichment of unknown microbiota. Our toolkit is available at https://github.com/lysovosyl/NanoDeep.

## INTRODUCTION

A nanopore sequencer is with an array containing thousands of sequencing units, which is composed of a reader, motor and tether protein; it determines the sequence of a target molecule (DNA, RNA or peptide) by analyzing the changes in electronic current (squiggle) when the molecule passes through the unit [1–3]. Oxford nanopore sequencers, such as MinION or PromethION, have recently been applied in biomedical research, especially clinical microbiological studies [4–6]. It provides several advantages in pathogen diagnosis and research compared with the next-generation sequencing (NGS) platform: (i) the sequencer is small and portable, appropriate for rapid diagnostics and fieldwork with limited laboratory conditions, which makes it possible for point-of-care testing (POCT) [7]; (ii) its sequencing length is longer than 2M, and the speed is higher than 450 nt/s, which enables analyzing microbial genome sequences in real time and in a high resolution [8, 9]; (iii) it can directly sequence the native DNA or RNA molecules without PCR amplification; and (iv) it simplifies the library preparation complexity and retains the native modification of the original molecules for downstream analysis [10–12]. However, some limitations restrict its applications on pathogen diagnosis [13, 14]: (i) the throughput is relatively low compared with the NGS platform [11]; (ii) the microbial DNA amount is extremely less than the host genomic content in clinical samples. Thus, improvements in the sequencing efficiency of microbial DNA molecules in a mixed sample enable its viability in pathogen diagnosis.

The genomic sequence of microbes can be enriched through biochemical methods, including lysis of host cells, PMA enrichment [15], PCR [16] and hybrid capture [17] of the sequences of interest; they require much more time, expertise, and equipment. These methods can enrich the pathogen genome to a degree, but the loss of targeted sequences in this procedure results in insufficient DNA material for further library preparation [18]. In contrast, a computational approach to enrich target sequences provides time, labor, and cost savings while reducing sample preparation complexity. Notably, the nanopore sequencer can reject a partially sequenced molecule by reversing the voltage across individually selected nanopores using ‘Read-Until’ utilities [19, 20]. Thus, it allows the selective sequencing of microbial sequences computationally by rejecting the unwanted sequences and enriches the microbial sequences of interest by analyzing the raw squiggles in real time.

Computational enrichment of target sequences holds an excellent advantage for clinical applications, but realizing this potential requires fast and accurate approaches for identifying molecules of interest because of the high speed of nanopore sequencing [21]. Recently, researchers made a great effort to develop computational approaches for microbial sequence enrichment incorporating ‘Read-Until’ utilities (Table S1). These methods are mainly in two classes: (i) the alignment-based method, which requires base calling or kmer mapping first and then determines the molecule of interest; (ii) the non-alignment-based method, mainly based on deep learning algorithms, which classifies signals directly by using the current signals (squiggles). The first approach requires converting a current signal into a base sequence using Guppy [22] or the representative kmer hash; thus, a large amount of computational resources is needed for the preprocessing step [23]. Meanwhile, the following alignment step requires a large genome index database and computational resources [18]. The representative solutions are Readfish, UNCALLED, ReadBouncer, BaseLess, metaRUpore, RawMap, RawHash, RUBRIC and BOSS-RUNS [20, 21, 24–30]. Notably, Shih *et al*. showed a lightweight mobile device embedded with a subsequence dynamic time warping (sDTW) algorithm achieved a good performance in adaptive sequencing, and Mikalsen *et al*. developed Coriolis embedded with a sequence alignment algorithm on a supercomputer-on-a-chip (SCoC) in adaptive sequencing [31, 32]. These approaches are based on sequence alignment or kmer mapping and thus limited by sequencing errors, reliance on genome indexes and inability to capture non-sequence information. The second approach directly binary classifies the signal into interest or non-interest sequences by analyzing the raw signal using a convolutional neural network (CNN) model [33], which requires extensive computational resources in training models but requires little resource in the application stage. SquiggleNet and DeepSelectNet demonstrated that the established CNN model enables the classification of microbial and host sequences at a higher speed compared with previous alignment-based methods [34, 35]; other works showed achievements in targeted sequencing under different biological contexts using a deep learning model, such as enrichment of noncoding RNAs, classifying microbial resistant genes and enrichment of mitochondrial genome [36–38]. However, further improvements in the deep learning-based methods are required for biomedical applications because (i) the biological rationale (the sequence composition or DNA modification) for the identification of microbial sequences using the CNN model is unclear; (ii) improvements in the speed and accuracy still benefit the enrichment of microbial sequences in clinical samples; and (iii) the published deep learning models require retraining the model in a new dataset and thus the robustness of the deep learning–based methods require further investigations.

Here, we first characterize the features of native microbial and human sequences through comparative analysis of nanopore sequencing signals and find that the kmer composition differs among them. To utilize this, we present a deep learning framework, NanoDeep, to overcome these limitations by incorporating CNN and squeeze and excitation (SE) [39]. We first showed that the raw squiggle derived from native DNA sequences determines the origin of microbial and human genomes. Then, we demonstrated that NanoDeep successfully classified bacterial reads from the pooled library with human sequence and showed enrichment for bacterial sequence compared with routine nanopore sequencing setting. Further, we showed that NanoDeep improved the sequencing efficiency and preserved the fidelity of bacterial genomes in the mock sample. In addition, NanoDeep performs well in the enrichment of metagenome sequences of gut samples, showing its potential applications in the enrichment of unknown microbiota.

## METHODS

### Microorganism culture

Seven bacterial strains have been included in our study, including *Escherichia coli DH5a, Staphylococcus epidermidis, Roseomonas mucosa, Pseudomonas aeruginosa, Staphylococcus hominis, Neisseria gonorrhoeae and Staphylococcus aureus*. *E. coli DH5a* was purchased from Tiangen (CB101-02). *S. epidermidis, R. mucosa, P. aeruginosa, S. hominis, N. gonorrhoeae* and *S. aureus* were kept by Dermatology Hospital, Southern Medical University. Six strains were cultured in LB medium with 200 rpm shaken overnight at 37°C except *N. gonorrhoeae*; *N. gonorrhoeae* was cultured in broth culture medium with 200 rpm shaken overnight at 37°C in a humidified atmosphere containing 5% CO_2_.

### Cell culture

Human embryonic kidney 293T cells (HEK 293T) were a gift from Prof. Bin Yang’s Lab. HEK293T were cultured in Dulbecco’s modified Eagle’s medium (DMEM; Gibco, C11995500BT) supplemented with 10% (v/v) FBS (BIOLOGICAL INDUSTRIES, 04-001-1A) and 1% (v/v) P/S (Gibco, 15140122). All cells were cultured at 37°C in a humidified atmosphere containing 5% CO_2_ and 95% air.

### Genomic DNA extraction

Genomic DNA was extracted from HEK293T cells with DNeasy Blood & Tissue Kit (Qiagen, 69504); microbial genomic DNA of *S. epidermidis, R. mucosa, S. hominis* and *S. aureus* were extracted with the QIAamp DNA Microbiome Kit (Qiagen, 51704); genomic DNA of other microorganisms was extracted by Rapid Bacterial Genomic DNA Isolation Kit (Sangon Biotech, B518225-0050). All procedures were carried out according to the manufacturer’s instructions. The high-molecular-weight DNA (>10 kb) was enriched through a low-concentrate agarose gel (0.6%) with TBE buffer (0.5%). DNA concentration was determined using the Qubit dsDNA HS (high- sensitivity) assay kit (ThermoFisher, Q32851); DNA purity was measured with the assessment curve shape and the ratio of OD 260/280 and OD 260/230 using NanoDrop. DNA fragment distribution was determined by Qsep100 (Bioptic) with S3 Cartridge (Kilo Base Cartridge, C105106). The fragment length information is shown in Figure S1 and Table S2.

### Construction of a metagenomic mock sample and nanopore sequencing

The mock sample was composited with equal amounts of DNA molecules (moles) of six microbial species (6 fmol, 2.08% per species), 3 fmol (1.04%) of *E. coli DH5a* and 250 fmol DNA molecules of the human genome (86.51%). Two-microgram DNA of the mock sample was subjected to library preparation using MinION with SQK-LSK110 Ligation Sequencing Kit (Oxford Nanopore). Subsequently, the data analysis showed that the mock sample consisted of 2.19% *S. epidermidis*, 0.44% *E. coli DH5a*, 0.84% *R. mucosa*, 1.92% *P. aeruginosa*, 3.18% *S. hominis*, 1.30% *S. aureus*, 1.37% *N. gonorrhoeae* and 75.57% *Homo sapiens*. There is a slight deviation in the data analysis result compared with the designed fraction because the measured DNA amount has a deviation in practice (Table S3).

### Deep learning model architecture

A CNN network has been applied in NanoDeep, which includes convolutional layers (CNNs), SE modules [39], residual blocks [40], AvgPool layers [41] and fully connected layers. The local features are extracted from the raw squiggles derived from nanopore MinION in the convolutional layer. Then, we weighted the features using SE modules, which can improve model performance by selectively emphasizing the important features. Subsequently, a three-layer residual module extracts deep-level features from the shallow features, weighted by an SE module. We further applied a Global Avgpool layer to improve computational efficiency by reducing the parameters and enabling NanoDeep to deal with signals of different lengths. Finally, a fully connected layer has been applied to generate a bi-classifier and output the probability for each class.

### 5-fold and leave-one-species-out cross-validation

The dataset was evenly divided into 5 folds. In each iteration of the 5-fold cross-validation, one of the folds is set aside as the validation dataset, while the remaining 4 folds are combined to form the training dataset. The model is trained using the training dataset and then evaluated using the validation dataset to assess its performance, including accuracy, precision and receiver operating characteristic (ROC) curve analysis. Each fold serves as the validation dataset once, ensuring that all folds are used as the training dataset at least once. In addition, we have performed leave-one-species-out cross-validation in such a manner that they exclude the DNA of one of the bacteria from the training and later test the performance of the model to classify the DNA signal of the excluded bacteria.

### Perform adaptive sampling using NanoDeep in real-time nanopore sequencing

The nanopore sequencing was done using the MinION device with FLO-MINI106 flow cell and SQK-LSK110 kit. The MinKNOW app (version 22.10.10) was utilized to obtain the sequencing data. To evaluate the host depletion function, we divided the whole flow cell into two sections: (i) channels 1–255 were dedicated to performing adaptive sampling using NanoDeep; (ii) channels 256–512 were left unused, and no specific operations were performed. Then, the sequence yield of the two sections is compared.

### Perform adaptive sampling on gut microbial sequencing on nanopore sequencing datasets

The nanopore sequencing dataset of gut microbiota in mice was downloaded from SRP219712 (Table S4). The dataset includes native DNA (NAT) samples and whole-genome amplified (WGA) of the same DNA sample. Each sample was divided into two parts: 80% of the data were used as training data, and the remaining 20% were allocated as validation data. We applied the training data of NAT samples to train the NanoDeep model and used the validation data from NAT samples and WGA samples to assess the performance of our model. Similarly, we evaluated the performance of NanoDeep in WGS samples with the same analysis.

## RESULTS

### Distinct 6mer electronic signal in the human genome compared with bacterial genomes

**Figure 1:**
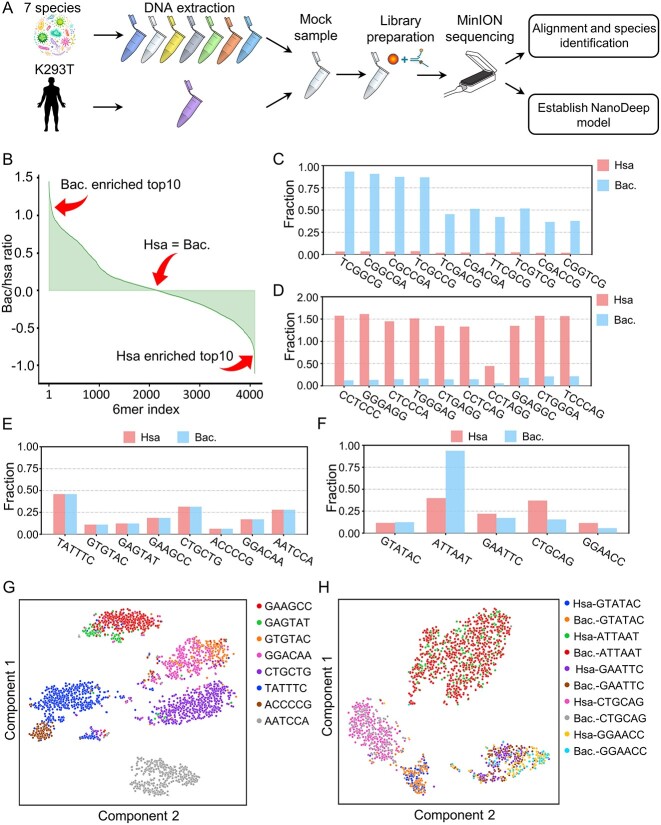
Distinct 6mer electronic signal in the human genome compared with bacterial pan-genome. (**A**) The scheme for nanopore sequencing on the mock sample using a single MinION flow cell. (**B**) The distribution of 6mers in the human genome compared with the bacterial genomes. (**C**, **D**) The top 10 enriched 6mers in human (C) and bacterial pan-genome (D). (**E**) The comparable 6mers in both human and bacterial genome. (**F**) The distribution of five 6mers commonly with DNA modification, such as 6 mA and 5mC. (**G**) Principal component (PC) analysis of electronic signal on 6mers, demonstrating the distinct electronic signal of 6mers and helpful for distinguishing human and bacterial sequences. (**H**) PC analysis of electronic signal on 6mers commonly with DNA modifications, showing that modification signal cannot distinguish human and bacterial sequences.

Nanopore adaptive sampling has been applied to the framework of nanopore sequencing using a deep learning computational model [42, 43]. However, the informative signal for detecting the difference between human and bacterial reads on nanopore adaptive sampling requires further investigation [44]. Here, we constructed a mock sample composed of HEK293T and seven bacterial strains and then subjected it to routine sequencing using a single MinION flow cell. The bulk sequencing data were obtained for downstream analysis (Figure 1A and Table S4). The raw reads were mapped to the human reference genome (hg38) and classified into bacterial using Kraken2. Then, we explored the composition and raw signal of 6mer derived from human and microbial sequences because ~6 nt of a DNA molecule reside in the pore when the measurements are taken [12, 45]. Interestingly, we found that the frequency distribution of 6mers in the human genome is distinct from the bacterial genomes in sequencing data and the reference genomes (Figure 1B–D); only a small fraction of 6mers is event distributed in the human and bacterial genomes (Figure 1E). It suggested that the sequence composition and its corresponding electronic signal are informative for distinguishing between human and bacterial reads. Furthermore, we found that the fraction of 6mers with frequent modification in bacteria are comparable, except ATTAAT, which is frequently with 6 mA modification. In addition, PCA analysis showed that a distinct electronic signal was observed in different 6mers (Figure 1G), but the electronic signal cannot distinguish 6mer (commonly with DNA modifications) in humans and bacteria (Figure 1H). In summary, the composition of 6mers and its adherent electronic signal are the keys to developing a model for performing nanopore adaptive sampling on distinguishing human and bacteria sequences.

### NanoDeep performs rapid adaptive sampling along nanopore sequencing

**Figure 2:**
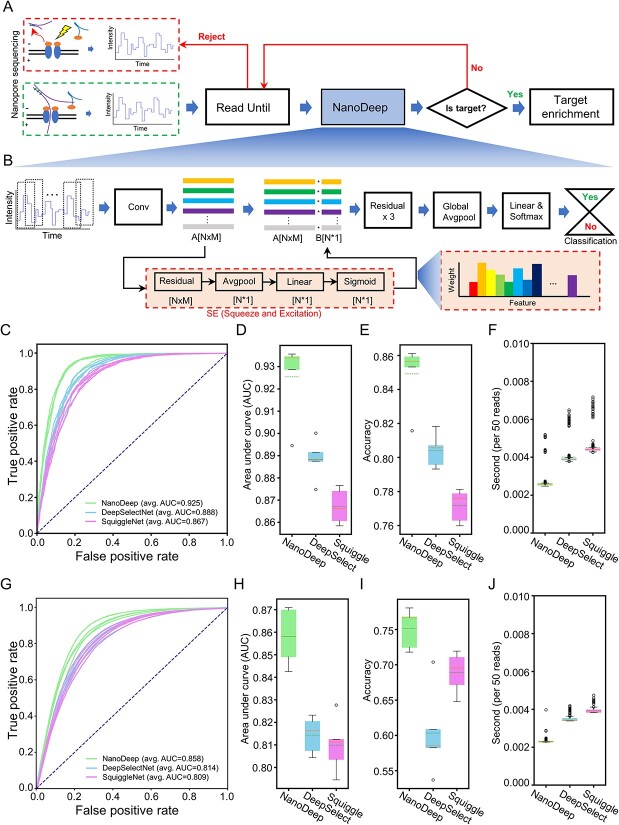
NanoDeep performs rapid adaptive sampling along nanopore sequencing. (**A**) The computational framework for nanopore adaptive sequencing. ReadUntil utility is used to obtain the raw electronic signal directly from nanopore sequencing in real-time. We developed NanoDeep to determine whether a fragment of the electronic signal is from the target reference. If the fragment is not from the target reference, a reject operation will be carried out by read until. (**B**) The construction of the deep neural network in NanoDeep algorithm; The training dataset is subjected to a CCN to extract informative signal block; then, SE is used to weigh the features and three-layer Residual is used for further construction of the prediction model; the Global AvgPool layers and fully connected layers form the final classifier; (**C**–**F**) The training and testing of NanoDeep, DeepSelectedNet and SquiggleNet models on the mock nanopore sequencing dataset. The performance of ROC curve (C), AUC (D), accuracy (E) and speed (F) is presented. (**G**–**J**) The performance of the NanoDeep, DeepSelectedNet and SquiggleNet models were trained with the simulated dataset on the nanopore sequencing dataset of the mock sample in our lab. The performance of the ROC curve (G), AUC (H), accuracy (I) and speed (J) is evaluated.

Previous studies showed the viability of nanopore adaptive sampling through sequence alignment and deep learning (CNN, LSTM, etc.) computational models [33, 34, 46]. However, the efficiency and high computing resource demand require further improvement. Here, we presented a computational framework for rapid adaptive sampling by integrating an innovative deep learning model NanoDeep and Read Until portal (Figure 2A and B). Briefly, the short raw electronic signals (~4000 signals in length) are obtained directly from MinION in real-time using Read Until; then, it determines whether a fragment of the electronic signal is from the target reference using NanoDeep. If the fragment is not from the target reference, a reject operation will be carried out to stop the sequencing through Read Until and a truncated sequence will be obtained; otherwise, the DNA fragment will continuously be sequencing, and the full length will be obtained (Figure 2A). Notably, the DNA sequence goes through the nanopore quickly; thus, it is important to classify the target sequence at a high speed. Given the observation of the distinct 6mer composition and electronic signal in nanopore sequencing, we developed NanoDeep to perform classification at high speed and with low computation resources through utilizing the CNN and SE module to extract localized features and select the emphasized important features. NanoDeep comprises several parts, including the CNN, SE module, three-tiered sequence of residual modules, Global AvgPool, and the fully connected layer (Figure 2B). Subsequently, we established the NanoDeep model using a mock sample and a stimulated dataset generated by DeepSimulator [47] that included 34 108 reads of humans and 34 108 of bacteria; it achieves accuracy and loss saturation with 30 epochs (Figure S2A and B). As a result, a 5-fold cross-validation analysis showed that the performance of NanoDeep is better than DeepSelectNet and SquiggleNet, which are neural network methods for nanopore adaptive sampling (Figure 2C–F, Figure S3A–D, Table S5). Notably, NanoDeep achieved a higher area under the curve (AUC) value of 0.925 and an accuracy score of 0.849 compared with DeepSelectNet (AUC: 0.888, Accuracy: 0.804) and SquiggleNet (AUC: 0.867, Accuracy: 0.771), indicating that our model has a better performance in distinguishing positive sequences from a large negative background (Figure 2D and E). On the other hand, our model processed 50 reads (~4000 signals, per read) using 2.89^*^10^−3^ s, which is a significant improvement compared with DeepSelectNet (4.05^*^10^−3^ s, per 50 reads) and SquiggleNet (4.43^*^10^−3^ s, per 50 reads) (Figure 2F), meaning that NanoDeep can handle and process a larger amount of data while maintaining performance. When we applied a NanoDeep model trained with a stimulated dataset in the nanopore sequencing dataset of the mock sample, we also observed better performance in AUC, accuracy and speed (AUC: 0.858, Accuracy: 0.752) compared with DeepSelectNet and SquiggleNet (Figure 2G–J and Table S5), suggesting that the trained NanoDeep model hold a good performance even applied in a new sequencing dataset. To test the robustness of the NanoDeep model, we applied the trained model to an independent dataset from Tourancheau *et al*. [41] and two nanopore sequencing datasets from two biological replicated experiments in our lab, indicating that it holds a stable performance in metagenomic NGS (mNGS) (Figure 5B) and tolerates the batch effects from different laboratory conditions (Figure S4). In addition, we performed cross-validation in a leave-one-species-out manner: the model was trained with the exclusion of one species, and the performance of the model was later tested to classify the raw signal of the excluded bacteria. As a result, NanoDeep can generalize well in most situations without the targeted species except *N. gonorrhoeae* (Figure S5A and B). In conclusion, we developed NanoDeep, which enables rapid adaptive sampling by incorporating a CNN and SE model.

### NanoDeep increases bacterial sequence yields in mock sample

**Figure 3:**
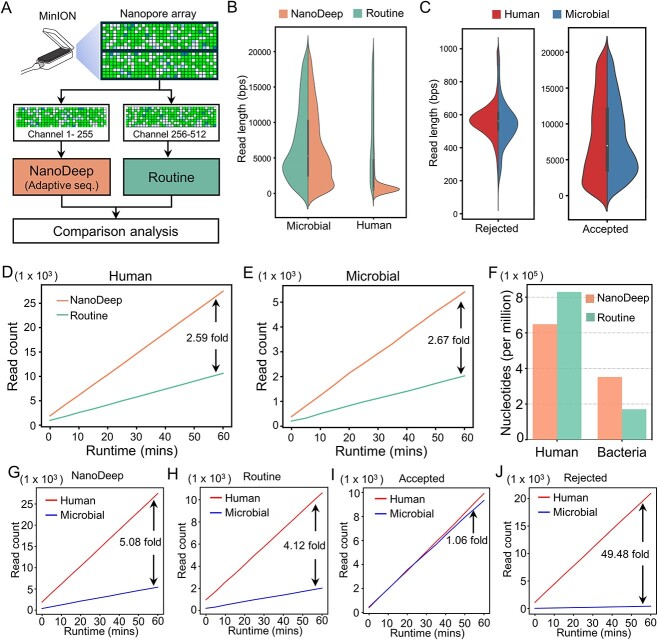
NanoDeep increases bacterial sequence yields in the mock sample. (**A**) The scheme for the experiment of the mock sample using MinION to test the performance of NanoDeep in real-time nanopore sequencing. (**B**) The read length distribution of microbial (left panel) and human-derived (right panel) sequences with and without adaptive sampling (NanoDeep). The violin diagram showed that the read length of human-derived sequences was enriched in ~600 nt upon adaptive sequencing, while the read length of microbial-derived sequences was similar in both adaptive sampling and the routine pipeline. (**C**) The read length distribution of human- and microbial-derived reads in the rejected and accepted read groups. (**D**) The count of the human-derived reads along NanoDeep adaptive sampling and routine sequencing mode. (**E**) The count of microbial-derived reads along NanoDeep adaptive sampling and routine sequencing mode. (**F**) The accumulated base per million of human- and bacteria-derived sequences. Those figures demonstrated that MinION produced a high yield upon nanopore adaptive sequencing using NanoDeep compared with routine pipelines. (**G**) The read count of human- and microbial-derived sequences along NanoDeep adaptive sampling. (**H**) The read count of human- and microbial-derived sequences along the routine sequencing. (**I**) The read count of human- and microbial-derived sequences in the accepted read group along NanoDeep adaptive sampling. (**J**) The read count of human- and microbial-derived sequences in the rejected read group along NanoDeep adaptive sampling.

Next, we designed an experiment to evaluate the performance of NanoDeep on the enrichment of bacteria sequences and depletion of human sequences in real-time nanopore sequencing. Briefly, we divided the sequencing nanopore array (512 nanopores) into two groups with the same number of channels: (i) channels 1–255 will be subjected to adaptive sampling using NanoDeep; (ii) channels 256–512 will be subjected to routine nanopore sequencing pipeline (Figure 3A). Therefore, we could unbiasedly compare the performance of NanoDeep and the routine pipeline. As a result, we performed nanopore sequencing in 1 h and collected 52 000 reads for further statistical analysis. We found that the human reads with a shorter length upon NanoDeep adaptive sequencing compared with the routine pipeline (Figure 3B, right panel), while the distribution of microbial read length is similar in NanoDeep and the routine pipeline (Figure 3B, left panel). In the meanwhile, we found that the length distribution of the rejected human and microbial reads is around 600 bps because a short fragment of the rejected sequence will be retained (Figure 3C, left panel), while the accepted human and microbial reads with a similar distribution (Figure 3C, right panel). This result showed that many human-derived reads were rejected, while only a few microbial-derived reads were rejected incorrectly in our experiment. Interestingly, we observed that the read count of both human and microbial is increased (~2.5-fold) upon NanoDeep adaptive sampling (Figure 3D and E, Table S6) and the cumulative base count of microbial genomes is higher in the NanoDeep adaptive sampling group compared with the routine group, suggesting that NanoDeep significantly enriches the microbial DNA sequences (Figure 3F and Table S6). Further analysis demonstrated that the cumulative read count and the human–microbial ratio are significantly increased in NanoDeep compared with the routine pipeline, indicating that the human sequences are successfully rejected and NanoDeep improves the sequencing efficiency (Figure 3G and H). Notably, we observed that the read count of human and microbial is comparable in NanoDeep and the routine pipeline, indicating that NanoDeep accepted a certain number of human sequences in error under a large negative background (Figure 3I). Remarkably, the human–microbial ratio of the rejected sequences achieved 49.48, suggesting a low rejection error for microbial sequences (Figure 3J). In addition, we obtained a similar performance of the NanoDeep model trained with the simulated dataset, suggesting the robustness of the NanoDeep algorithm (Figure S6A–I). In summary, the NanoDeep model efficiently rejects the human-derived sequence and increases the sequencing yields of the microbial DNA sequences in nanopore read-time sequencing, suggesting its potential applications in genomic studies.

### NanoDeep enables the unbiased recovery of seven bacterial genomes

**Figure 4:**
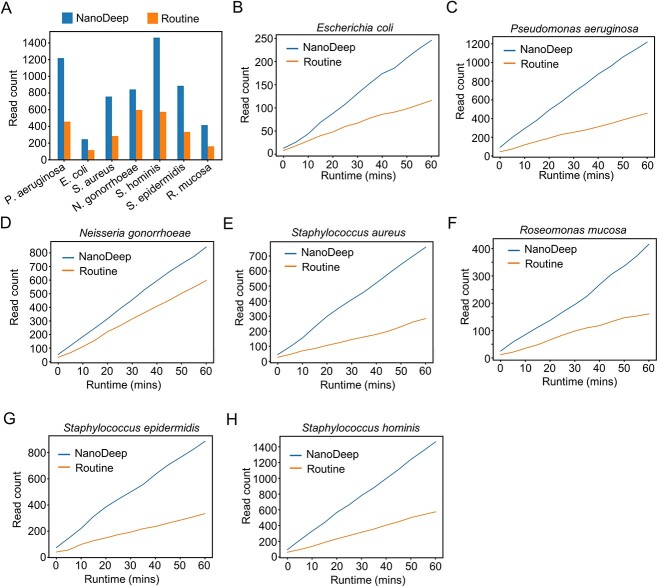
NanoDeep recovers the fidelity of bacterial pan-genomes. (**A**) The read count of seven bacteria using NanoDeep adaptive sampling and routine sequencing mode; the bar chart showed a significant increase of read count in seven species upon NanoDeep adaptive sampling compared with the routine pipeline. (**B**–**H**) The read count of seven bacteria along NanoDeep adaptive sequencing, including *E. coli* (B), *P. aeruginosa* (C), *N. gonorrhoeae* (D), *S. aureus* (E), *R. mucosa* (F), *S. epidermidis* (G) and *S. hominis* (H) along with 1 h nanopore sequencing; the line chart showed that NanoDeep recovered the abundance and fidelity of seven species in the predefined mock library.

NanoDeep can efficiently reject the reads derived from the host genome, but whether it could recover the fidelity of the targeted bacterial genome requires further investigation. Therefore, we sought to investigate the characteristic of reads in different species. As a result, we found that the read count of six bacterial genomes shows at least 2-fold enrichment with adaptive sampling using NanoDeep compared with the routine sequencing mode except *N. gonorrhoeae* (~1.4-fold), double confirming that NanoDeep not only efficiently rejects the human reads but also retains reads derived from bacterial genomes (Figure 4A, Table S6). Further, we found a similar read count distribution of seven species along nanopore sequencing, indicating that NanoDeep recovers the fidelity of the bacterial genomes (Figure 4B–H and Figure S7). Notably, we observed a similar fold of enrichment in *E. coli, P. aeruginosa, N. gonorrhoeae, S. epidermidis, S. hominis, R. mucosa* and *S. aureus* upon NanoDeep adaptive sampling compared with the routine mode, suggesting that NanoDeep unbiasedly obtained the genomic sequences of seven bacteria, while depleted the human sequences in the pooled library. In conclusion, our analyses demonstrate that NanoDeep efficiently rejects the target sequence and recovers the fidelity of bacterial genomes.

### NanoDeep performance on mouse gut microbiota sequencing

**Figure 5:**
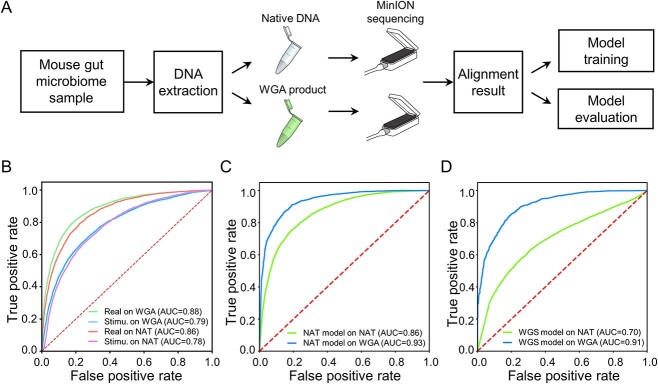
NanoDeep application on gut microbiota sequencing. (**A**) The pipeline for nanopore sequencing on mouse gut microbiome, data processing and the model training. (**B**) The ROC analyses of NanoDeep on the nanopore sequencing data derived from the NAT sequencing and WGA DNA sequencing. The curve showed that the pre-trained model using the stimulated dataset came up with an AUC less than 0.8 in both NAT and WGA samples, while the pre-trained model using the mock dataset came up with an AUC greater than 0.85 in both NAT and WGA samples. (**C**) The ROC analyses of NanoDeep on the nanopore sequencing data derived from the NAT sequencing and WGA DNA sequencing with the model trained by the nanopore sequencing data derived from NAT DNA of the mouse gut microbiome sample. (**D**) The ROC analyses of NanoDeep on the nanopore sequencing data derived from the NAT sequencing and WGA DNA sequencing with the model trained by the nanopore sequencing data derived from WGA DNA of the mouse gut microbiome sample.

Previous reports showed that the deep learning model outperforms the alignment-based method in nanopore adaptive sampling [34]. However, the performance of a trained NanoDeep model on a new application warrants further exploration. To this end, we obtained a published dataset including the nanopore sequencing data derived from the NAT and WGA DNA in mouse gut microbiome and subjected to standardized species classification for further investigations (Figure 5A and Table S4). We first applied two pre-trained models with the simulated and mock datasets to distinguish the host and bacterial reads in WGA and NAT samples. As a result, ROC analyses showed that the model trained with the stimulated dataset achieved an AUC value of 0.78 in the NAT sample and 0.79 in the WGA sample, while the model trained with the mock dataset achieved an AUC value of 0.86 in the NAT sample and 0.88 in the WGA sample (Figure 5B), indicating that the pre-trained model can transfer to other applications and with a moderate performance in depletion of the host genomic sequences. Interestingly, we achieved a better performance if we applied NanoDeep to WGA samples (0.93 and 0.91) using the model trained by the nanopore sequencing data derived from the NAT or WGA DNA in mouse gut microbiome (Figure 5C and D, blue lines), but the model trained with NAT or WGS sample did not perform well in the NAT sample (Figure 5C and D, green lines). We suspected that the native DNA commonly has modification and the signal will be perturbed with noise. Thus, a large sample size was required to achieve a higher accuracy using NanoDeep in further applications. In summary, NanoDeep provides an alternative way to perform adaptive sampling in a library with abundant host DNA sequences; it rapidly obtains genomic information and improves the sequencing yields of the microbial genomic sequences.

## DISCUSSIONS

Nanopore sequencing technology has been applied in recent biomedical research, but the low throughput and sequencing accuracy limit its application in clinical diagnosis and field research [48–50]. The high abundance of host nucleotide contamination significantly impacts pathogen diagnosis [51]. Notably, the Oxford nanopore sequencer provides a ReadUntil toolkit for selective sequencing the DNA/RNA molecules of interest through reversing the voltage across individually selected nanopores [18, 20]; the computational framework for analyzing sequencing squiggles and determining the sequences of interest in real time is required for further investigations [52]. Here, we develop NanoDeep to determine the sequences of microbial and human through incorporating CNN and SE models. NanoDeep successfully classifies bacterial reads from the pooled library with human sequences and shows enrichment for bacterial sequences compared with routine nanopore sequencing. It also performs well in the enrichment of metagenome sequences of gut samples, indicating its potential applications in the enrichment of unknown microbial sequences.

Even though a deep learning model has been applied to distinguish microbial from human sequences, the sequence itself or the chemical modifications within the sequences that contribute to the predictive power remain unknown. Previous studies showed the abundance of different chemical modifications in microbial and mammal genomes, which helps classify sequences derived from microbial and mammal genomes [34]. Interestingly, we observed the differences in electronic current signals with or without chemical modifications among different species in our initial analysis, but it did not help distinguish different species. We found that the composition of 6mers is different in microbial genomes compared with mammal genomes. It suggests that the computational model and the signal features of the 6mers composition may be helpful for species classification. Therefore, we incorporate the CNN and SE to extract the features of local kmers within the read-time squiggles. Notably, we trained the model using the simulated WGS dataset; the model can distinguish the signal differences between microbiota and mammalian. Thus, incorporating simulated data for the training process of the NanoDeep model is helpful for real-world applications.

The ideal model for microbial adaptive sampling should satisfy the following criteria: (i) the speed for classifying a batch of reads should be faster than the sequencing speed of a nanopore sequencer (450 nt/s); (ii) discriminate the human and microbial sequences in high accuracy; and (iii) recover the fidelity of the genomic composition of the sequencing library. NanoDeep achieved better speed and accuracy performance than DeepSelectNet and SquiggleNet. The later analysis showed that NanoDeep recovered the species composition of the mock sample, indicating that it meets the requirement for adaptive sampling in nanopore platforms. Notably, the enrichment of *N. gonorrhoeae* is relatively lower than that of other species under NanoDeep adaptive sampling (Figure 3A, Table S6). Further cross-validation analysis in a leave-one-species-out manner shows a similar result using the model trained with or without *N. gonorrhoeae* (Figure S5). We suspected that the composition of the sequence library may slightly affect the performance of our model. Further incorporating other characteristics of microbial sequences may enable a better performance of NanoDeep.

Previous deep learning models applied to microbial adaptive sampling always require retraining models in different situations; thus, the robustness of the deep learning-based methods requires further investigation. Our study showed that the accuracy and loss of the NanoDeep model become saturated within 30 epochs in the training process (Figure 2A and B); NanoDeep exhibited a better performance in speed and accuracy than the existing deep learning model. We reasoned that the NanoDeep model may be suitable for microbial enrichment in different situations because it utilizes the 6mer characteristics shared with microbial species but distinct from mammalian genomes. As expected, NanoDeep maintains its performance in other datasets from our lab or the published works, only with a slight decrease in AUC and accuracy. Further, training a NanoDeep model with a broader range of species may help improve the performance of adaptive sampling.

In conclusion, our study tries to understand the fundamental principles underlying nanopore sequencing signal classification using a deep learning model. Then, we devised NanoDeep to leverage simulated data for training the model and implementing it in real-world sequencing scenarios. Our method successfully enriches the sequences derived from the target species in the simulated dataset, mock sample and metagenome sequences of gut samples. However, it is worth noting that our model currently exhibits a relatively lower accuracy when transferring NanoDeep to different biological contexts. Further improvements in the accuracy of our prediction model will ensure the potential application of pathogen diagnosis.

## CONCLUSIONS

Deep analysis of the raw squiggles shows that the signal of 6mers in microbial differs from mammal genomes in nanopore sequencing. Upon these observations and the corresponding design of a deep-learning framework, NanoDeep successfully enriches the microbial sequences through real-time depletion of human sequences in the mock samples and real-world applications. NanoDeep achieves a good performance only with the stimulated nanopore sequencing data and performs well in host sequences depletion in metagenomic sequencing. Therefore, NanoDeep has a wide range of applications in the biomedical research field.

**Key Points**

- The signal of 6mers in microbial differs from mammal genomes in nanopore sequencing.
- NanoDeep trained with stimulated nanopore sequencing dataset achieved high accuracy in real-world nanopore application.
- NanoDeep successfully enriches the bacterial sequences through real-time depletion of human sequences in the mock sample.
- NanoDeep enables unbiased recovery of most species in the mock sample and is suitable for microbial enrichment in metagenomic sequencing.

## Supplementary Material

Suppl_figures_v4_bbad499

Supplementary_Tables_v4_bbad499

## Data Availability

NanoDeep package and the trained models are available on GitHub: https://github.com/lysovosyl/NanoDeep. The nanopore sequencing dataset used in this study has been deposited in NCBI SRA databases with accession number SRP449339. The nanopore sequencing dataset of gut microbiota in mice was downloaded from SRP219712.

## Author Biographies

**Yusen Lin** is a Research Assistant fellow at the Dermatology Hospital, Southern Medical University, working on microbial identification and RNA biology using deep learning algorithms.

**Yongjun Zhang** is a PhD candidate at Dermatology Hospital, Southern Medical University. His research focuses on skin biology and new NGS applications in diagnosis.

**Sun Hang** is a Research Assistant at Dermatology Hospital, Southern Medical University. He works on method development for high-throughput sequencing.

**Jiang Hang** is an MPhil candidate at Dermatology Hospital, Southern Medical University. His research focuses on the mechanistic investigation of noncoding RNAs.

**Xing Zhao** is a PhD candidate at Li Ka Shing Institute of Health Sciences, The Chinese University of Hong Kong. He works on developing targeted sequencing methods using deep learning and nanopore sequencing.

**Xiaojuan Teng** holds an MPhil degree from Dermatology Hospital, Southern Medical University. She works on the functions of long noncoding RNAs in aging.

**Jingxia Lin** is a Research Assistant at Dermatology Hospital, Southern Medical University. Her research focuses on developing innovative sequencing technologies formolecular diagnosis.

**Bowen Shu** is an associate professor at the Dermatology Hospital, Southern Medical University. He works on innovative technologies for pathogen diagnosis using microfluid and biochemistry.

**Hao Sun** is a Full Professor at the Li Ka Shing Institute of Health Sciences (LiHS) and the Department of Chemical Pathology, Chinese University of Hong Kong. He is working on understanding the fundamental aspects of transcriptional regulation of both coding and noncoding RNAs using integrated genomic and bioinformatics approaches.

**Yuhui Liao** is the Chief Scientist of the National Key R&D Program and works at the Dermatology Hospital at Southern Medical University. He works on constructing high-sensitivity functional nucleic acid probes and the quantitative analysis of makers in infection diseases.

**Jiajian Zhou** is an associate professor at the Dermatology Hospital, Southern Medical University. His research focuses on understanding the behavior of RNA/DNA molecules using large-scale data analysis and deep learning algorithms.
