# Supplementary material for: NanoDeep: a deep learning framework for nanopore adaptive sampling on microbial sequencing
Source: Brief Bioinform. 2024 Jan 6;25(1):bbad499. doi: 10.1093/bib/bbad499 (PMC10772945; doi:10.1093/bib/bbad499)
Supplement: Suppl_figures_v4_bbad499 [file suppl_figures_v4_bbad499.pdf]

Figure S1.

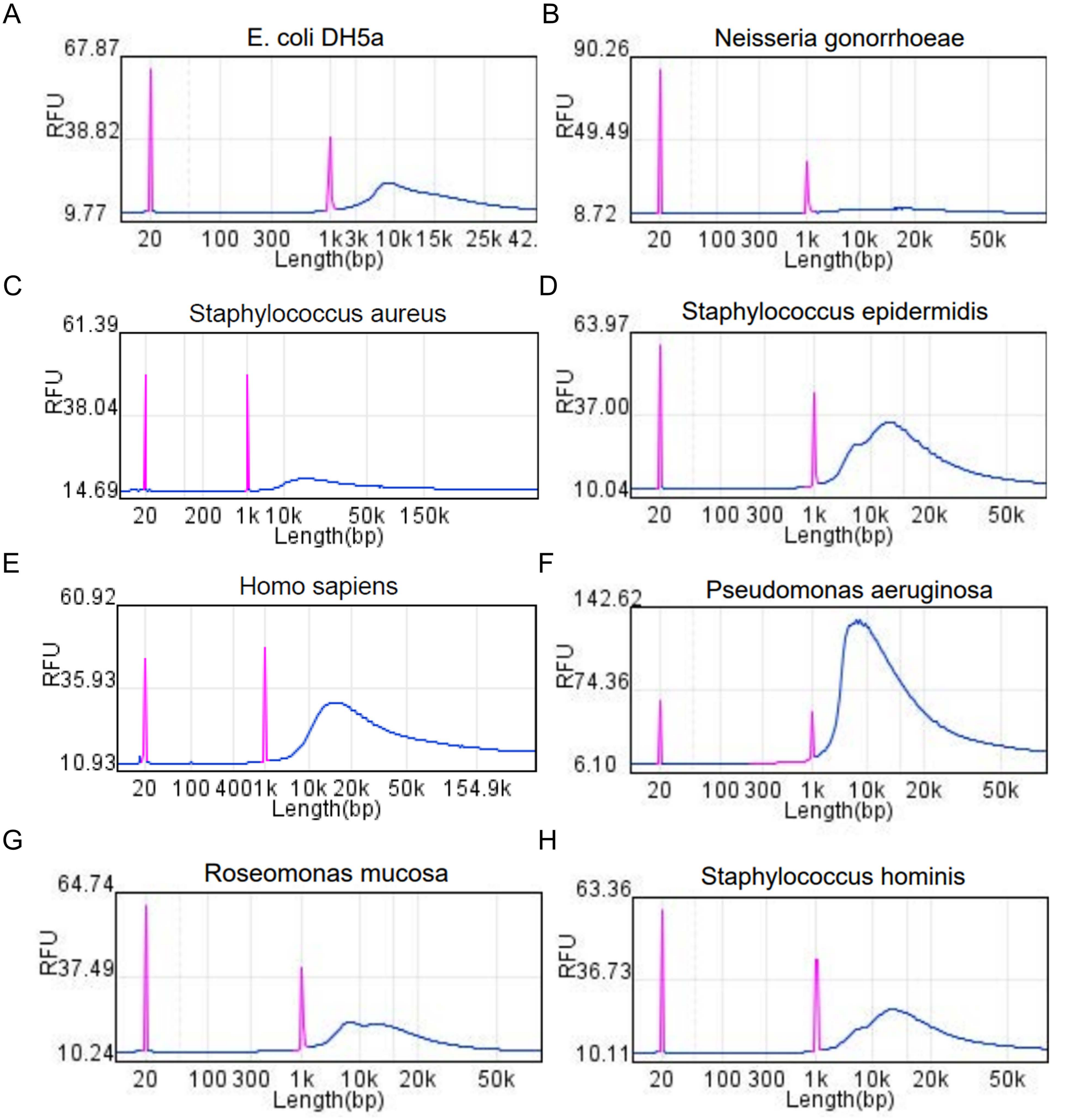

Figure S1. The fragment analysis of the extracted DNA derived from 8 species for nanopore sequencing. *Escherichia coli (A), Neisseria gonorrhoeae (B), Staphylococcus aureus (C), Staphylococcus epidermidis (D), Homo sapiens (E), Pseudomonas aeruginosa (F), Roseomonas mucosa (G), Staphylococcus hominis (H).* RFU, relative fluorescence units.

Figure S2.

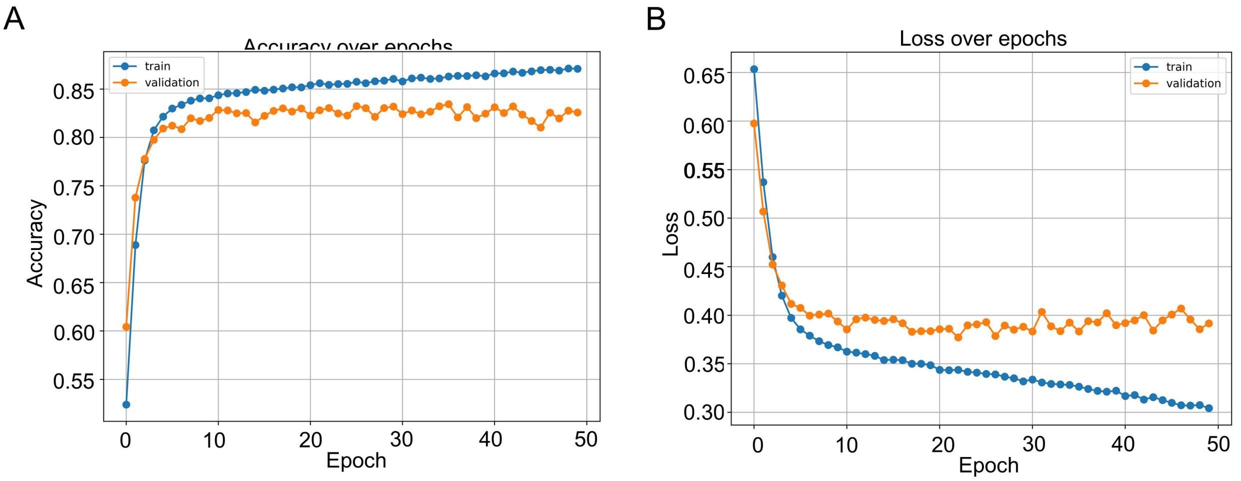

Figure S2. The accuracy and loss over epochs in the training process of the NanoDeep model. A. line plot shows the accuracy reach saturation after 30 epochs; B. line plot shows the loss reach saturation after 30 epochs.

Figure S3.

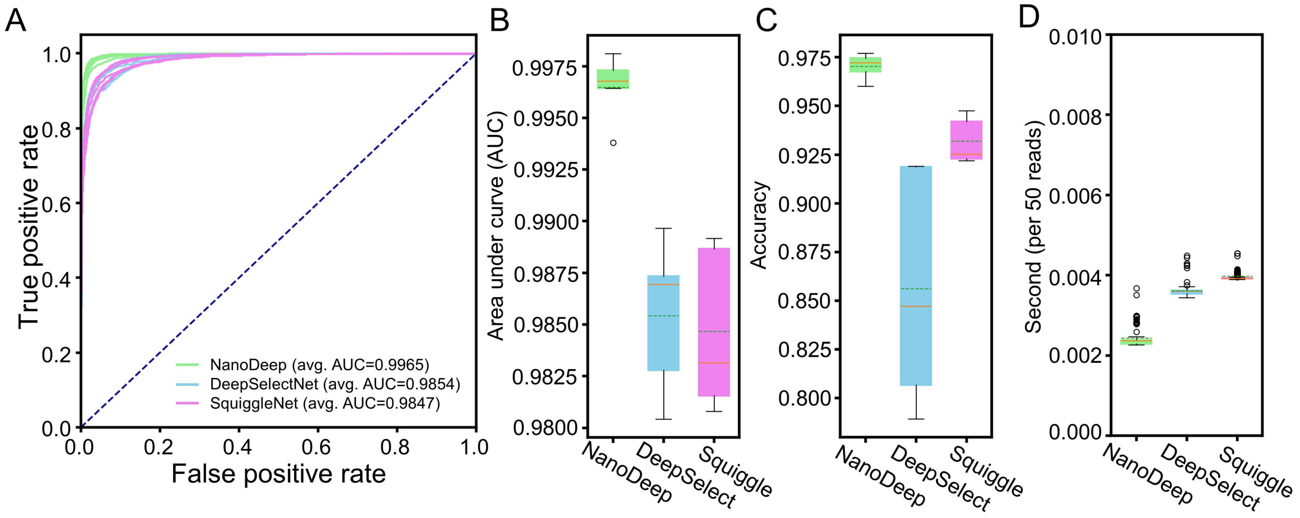

Figure S3. The performance of NanoDeep on the stimulated nanopore sequencing dataset with the model trained with the same dataset. A-D. The performance of the Receiver Operating Characteristic (ROC) curve (A), Area Under Curve (AUC) (B), accuracy (C) and speed (D) is evaluated.

Figure S4.

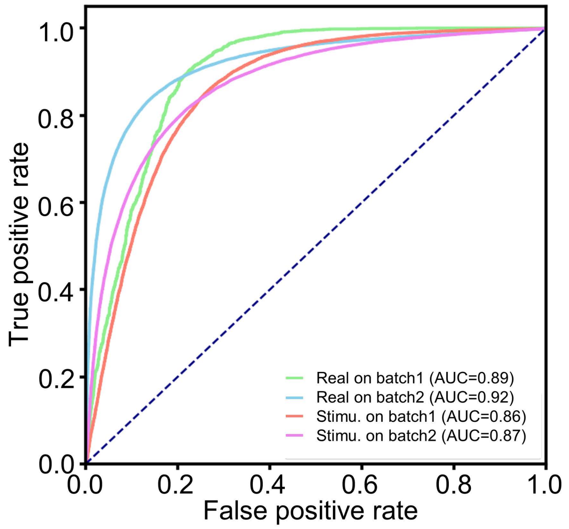

Figure S4. The robustness of the NanoDeep model. ROC curves show that the NanoDeep model trained with read-world (Real) and stimulated (Stim.) data performed well on two independent datasets from our lab: batch1 represents the nanopore sequencing dataset derived from an experiment done on 2023.04.24 (SRR25258892); batch2 represents the nanopore sequencing dataset derived from an experiment done on 2022.11.27 (SRR25423905).

Figure S5.

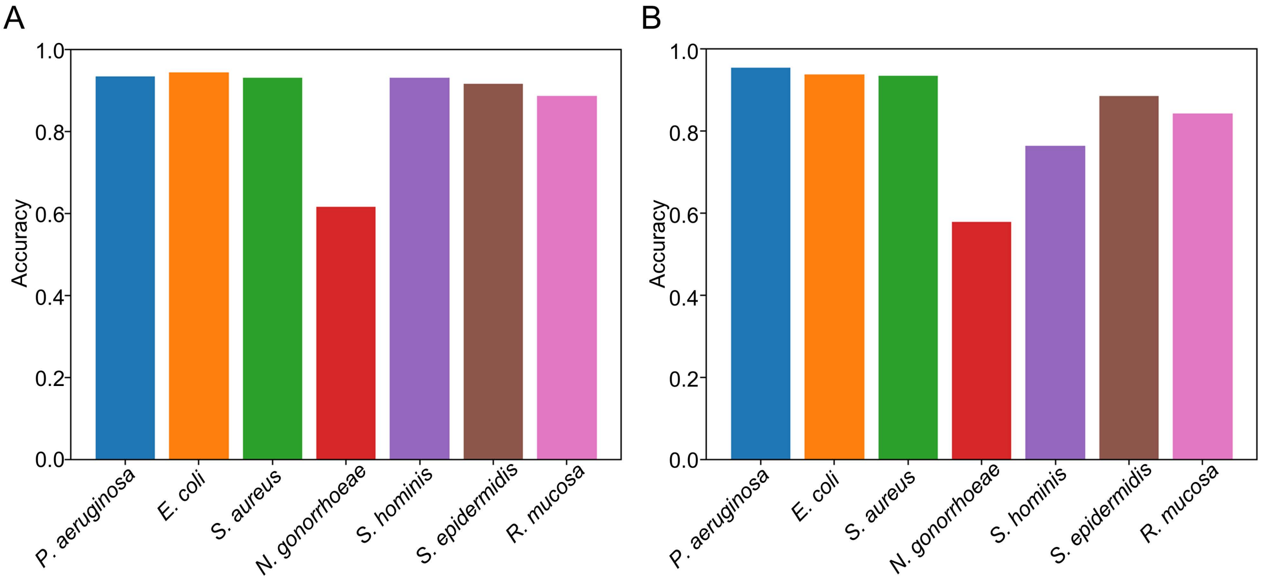

Figure S5. The performance of NanoDeep with various genomics compositions in the mock dataset. A. the bar chart shows that NanoDeep achieved an accuracy greater than 0.9 in 6 species except *Neisseria gonorrhoeae* using the model trained with a dataset including seven bacteria and human reads; *B.* The bar chart shows that NanoDeep achieved a similar accuracy in 6 species except *Neisseria gonorrhoeae* using the model trained with a dataset excluding the reads from the targeted species. These results suggest that NanoDeep performs well regarding genomic composition in applying microbial read enrichment.

Figure S6.

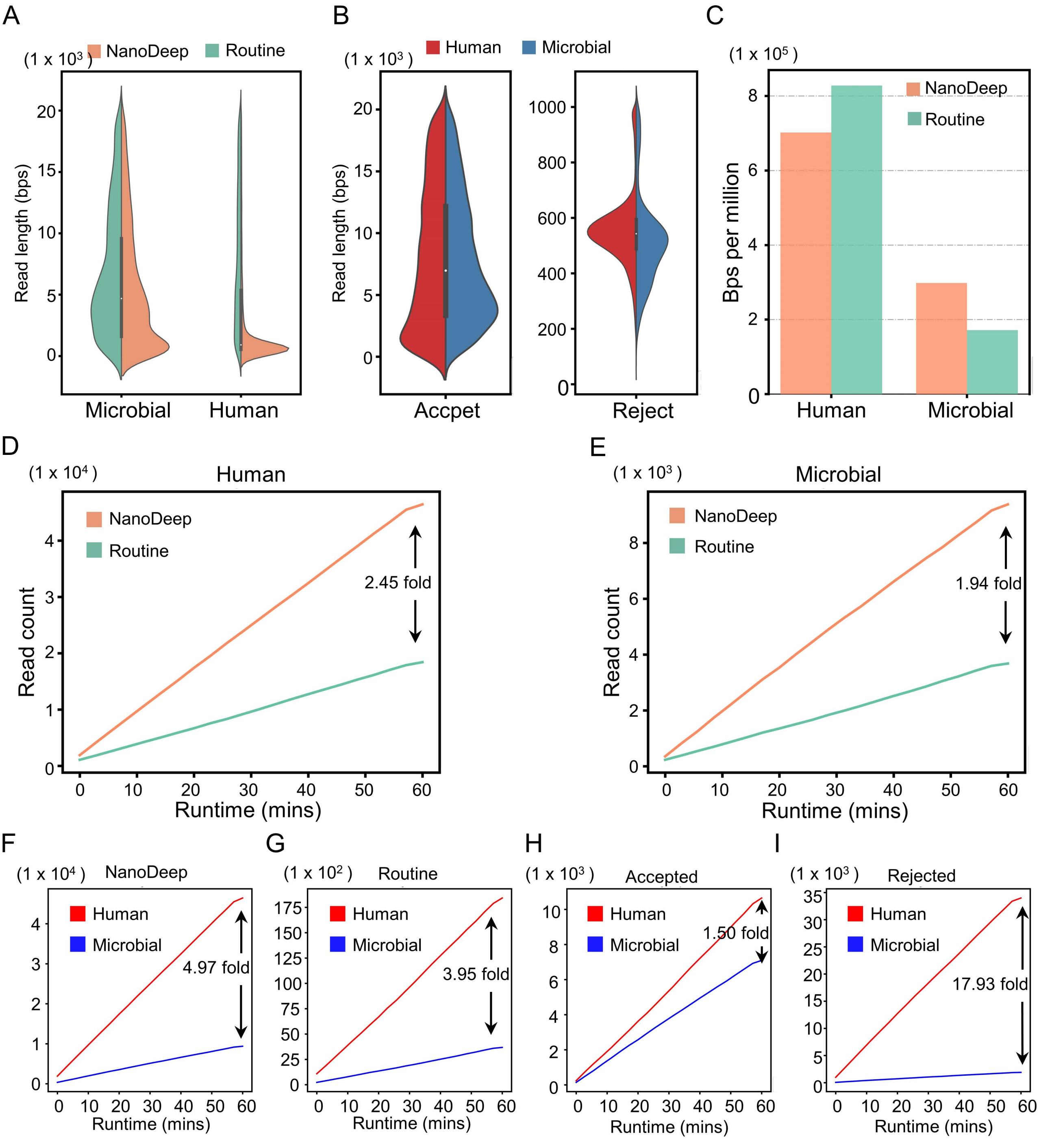

Figure S6. NanoDeep trained with the simulated data increases microbial sequence yields in the mock sample. A. The read length distribution of human- and bacterial-derived sequences with and without NanoDeep adaptive sampling. The violin diagram showed that the read length of human-derived sequences was enriched in 600 nts upon adaptive sequencing, while the read length of microbial-derived sequences was identical in both adaptive sampling and routine sequencing mode; B. The reads length distribution of human- and microbial-derived sequences in adaptive sampling in both the accepted and rejected groups; C. The accumulated base per million human- and microbial-derived sequences; D and E. The count of human- and microbial-derived reads in adaptive sampling and routine sequencing modes; F and G. The count of human- and microbial-derived reads in adaptive sampling (F) and routine sequencing (G); H and I. The count of human- and microbial-derived reads in adaptive sampling in the accepted (H) and rejected (I) group.

Figure S7.

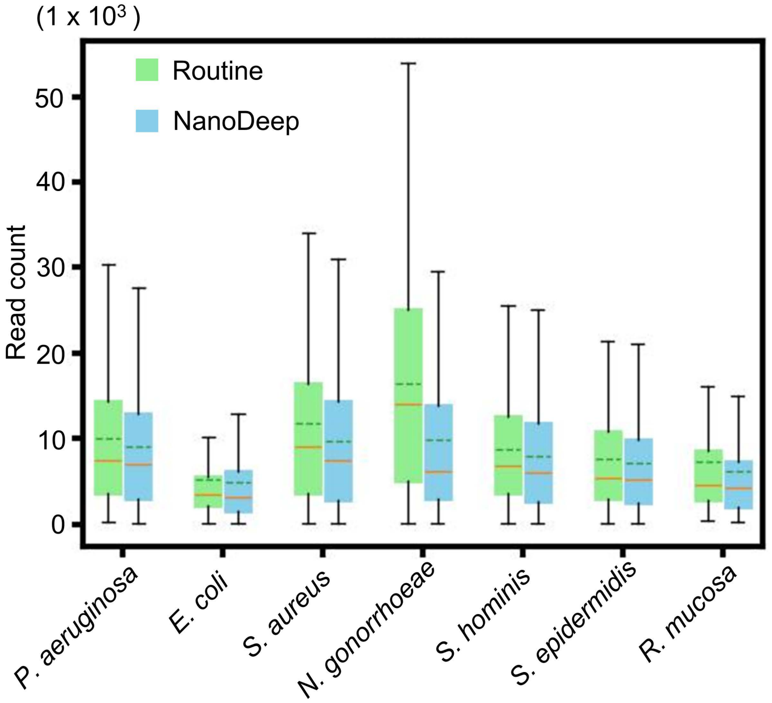

Figure S7. The read count distribution of different microbial species in NanoDeep adaptive sequencing and routine sequencing. It shows that microbial read count in NanoDeep adaptive sequencing is comparable with Routine sequencing across seven species.
